# Supplementary material for: Novel Sonoguided Digital Palpation and Ultrasound-Guided Hydrodissection of the Long Thoracic Nerve for Managing Serratus Anterior Muscle Pain Syndrome: A Case Report with Technical Details
Source: Diagnostics (Basel). 2025 Jul 28;15(15):1891. doi: 10.3390/diagnostics15151891 (PMC12346195; doi:10.3390/diagnostics15151891)
Supplement: Supplementary file 1 [file diagnostics-15-01891-s001.zip › diagnostics-3648780-supplementary.pdf]

Video S1 link:
https://www.dropbox.com/scl/fi/e9lwplf3oz1anz5ui59sj/LTN-SDP-with-illustration.mp4?rlkey=vv989i4e26anfxl07cnn35jcl&dl=0 (accessed on Jul 27, 2025).

Video S1 Dynamic sonographic visualization of serratus anterior anatomy during Sonoguided Digital Palpation (SDP), demonstrating: (1) normal sonographic architecture of the serratus anterior muscle (SAM) and fascia (SAF), (2) pathological long thoracic nerve (LTN) morphology, and (3) reproduction of concordant pain through targeted LTN palpation, confirming serratus anterior muscle pain syndrome (SAMPS) diagnosis.

Video 2 link:
https://www.dropbox.com/scl/fi/llvwky7vdnnv1aevtqnle/LTN-HD-7-labeled-with-sonoantomy.mp4?rlkey=34y01902km2qzkevaybw1i33u&dl=0 (accessed on Jul 27,29025)

Video S2: Ultrasound-guided hydrodissection of the long thoracic nerve using 20 mL of 5% dextrose water (D5W) without local anesthesia. Key anatomical structures visualized: long thoracic nerve and lateral thoracic artery (LTN & LTA), serratus anterior muscle and fascia (SAM, SAF), latissimus dorsi muscle and fascia (LD, LDF), thoracodorsal nerve and artery (TDN&A), teres major muscle (TMa), and intercostal muscle (ICM).
